# The contribution of the ABCG2 C421A polymorphism to cancer susceptibility: a meta-analysis of the current literature

**DOI:** 10.1186/1471-2407-12-383

**Published:** 2012-09-01

**Authors:** Pin Chen, Lin Zhao, Peng Zou, Haitao Xu, Ailin Lu, Peng Zhao

**Affiliations:** 1 Department of neurosurgery, the First Affiliated Hospital, Nanjing Medical University, Nanjing, China; 2 The First Affiliated Hospital of Nanjing Medical University, 300 Guangzhou Road, Nanjing, 210029, China

## Abstract

**Background:**

ABCG2, also known as BCRP, is a half ATP-binding cassette (ABC) transporter that localizes to plasma membranes. Recently, a number of studies have investigated the relationship between the C421A polymorphism in ABCG2 and cancer risk in multiple populations and various types of cancers; however, this relationship remains unclear. Therefore, we performed a meta-analysis to further explore this association.

**Methods:**

The meta-analysis incorporated 10 studies involving a total of 3593 cases and 5875 controls. Odds ratios (ORs) and 95% confidence intervals (CIs) were calculated based on the date extracted from the studies to evaluate the strength of association. We also analyzed the heterogeneity and sensitivity of each report and the publication bias of the studies.

**Results:**

Overall, our results showed that there appeared to be a significant association between the ABCG2 C421A polymorphism and decreased cancer susceptibility (heterozygote-AC versus CC: OR = 0.759, 95%CI = 0.620-0.930; dominant effects model-AA/AC versus CC: OR = 0.771, 95%CI = 0.634-0.938; additive effects model-A allele versus C allele: OR = 0.809, 95%CI = 0.687-0.952). Similarly, decreased cancer risk was also found after stratification of the SNP data by cancer type, ethnicity and source of controls in heterozygote model, dominant effects model and additive effects model.

**Conclusions:**

We found that the ABCG2 C421A polymorphism is a protective factor for developing cancer. The same relationship was found when the studies were stratified by cancer type, ethnicity and source of controls.

## Background

Cancer is a multi-factorial disease, which results from complex interactions between environmental and genetic factors, has become one of the most challenging health issues today [1]. In contrast to environmental variables, the genetic variables ranging from single-nucleotide substitutions to major chromosomal aberrations may make more contribution to the cancer development [2,3]. In the last few years, an increasing number of studies have been conducted to assess the relationship between the hereditary factors and cancer risk.

ABC transporters, or the family of adenosine triphosphate (ATP) binding cassette are a large superfamily of transmembrane glycoprotein and contain over 50 members which can mediate the transfer of a wide variety of substrates across cellular membranes [4]. ATP-binding cassette G2 (ABCG2), originally known as Breast Cancer Resistant Protein (BCRP), first discovered in doxorubicin-resistant breast cancer cells is a major member of the ATP-binding cassette transporter family and located on chromosomal region 4q22 encoding a 72-kDa membrane protein [5,6]. It is constitutively expressed in various tissues such as the embryonic stem cells, placental syncytiotrophoblasts, pancreas, liver, gastrointestinal, muscle and immature hematopoietic tissues [7-9]. Previous functional researches reviewed that ABCG2 can transport a wide spectrum of substrates, ranging from chemotherapeutic agents to carcinogenic xenobiotics [10-13]. ABCG2 may play an important role in controlling the cellular export of xenobiotic molecules. The differential metabolism of xenobiotics due to variations in the transporter molecules may affect the risk of some cancers [14-18].

It has been recognized that single nucleotide polymorphisms (SNPs) are the most common inherited sequence variations in the human genome. These polymorphisms can change the expression and activity of the corresponding genes and their proteins and affect the susceptibility to different types of cancers [19-23]. Researches had shown that there were two frequently polymorphic SNPs in the BCRP gene: one in exon2 (G34A, resulting in a V12M change) and the other in exon5 (C421A, resulting in a Q141K substitution) respectively [15]. The C421A polymorphism (rs2231142) in ABCG2 which lead to a glutamine-to-lysine amino acid substitution is found at different frequencies in different ethnic populations (Asians: 35%, Caucasians: 10%) and is apparently correlated with the reduced expression and activity of BCRP protein [21,24-26].

To date, numerous studies have investigated the association between the C421A polymorphism in ABCG2 and the cancer susceptibility. Unfortunately, however, the results of these studies have been inconsistent. Therefore, in order to gain insights into the association between polymorphism of ABCG2 C421A and cancer risk, we conducted a meta-analysis of eligible case–control and cohort studies.

## Methods

### Study eligibility and validity assessment

We conducted a systematic search in PubMed and Embase (last updated on February 10, 2012) using the terms "(ABCG2 or BCRP) and polymorphism "without any restriction in language and publication year. To identify other relevant studies, the articles cited by the retrieved studies were also searched. Each of the selected articles in our meta-analysis met all of the following criteria: 1) the article pertained to the ABCG2 C421A polymorphism and cancer risk; 2) the design was a human case–control or case-cohort study; and 3) the genotype frequencies in the cancer cases and controls were available. Studies were excluded if they did not include a control population, did not determine genotype frequency and or were duplicates of previous publications.

### Data extraction

The data were extracted from all eligible studies that met the selection criteria listed above. The data extraction was performed by two authors independently and any disagreements were resolved by discussion between the two authors. The data collected from each study were as follows: the first author's name, publication year, country of origin, ethnicity, cancer type, control groups source, number of cases and controls, and genotype frequencies for cases and controls. The deviation of the genotype frequencies in the control populations from Hardy-Weinberg Equilibrium (HWE) was calculated separately for each study. The control group sources were classified as population-based controls, hospital-based controls or mixed (both population- and hospital-based) controls. The population ethnicity was classified as Asian or Caucasian.

### Statistical analysis

We conducted all statistical analyses using STATA software (version 11; Stata Corporation, College Station, Texas). All P-values were two-sided and P < 0.05 was considered statistically significant. Odds ratios (ORs) and 95% confidence intervals (CIs) were calculated to assess the strength of the association between the ABCG2 C421A polymorphism and the cancer risk. We also examined the overall association of the A allele of C421A with risk of cancers and compared cancer incidence in homozygote (AA versus CC) model and heterozygotes (AC versus CC) model, dominant (AC/AA versus CC) model and the recessive (AA versus CC/AC) model. The values for ORs and CIs of each individual were considered twice. Stratified analyses were conducted by cancer type, source of controls and ethnicity. The Hardy–Weinberg equilibrium was calculated for all the control groups of each study, and control groups of studies that were not in HWE (p < 0.05) were excluded. A chi-squared-based Q-statistic test was used to detect the heterogeneity among studies. When the P-value of the Q-test was > 0.05, which indicated a lack of heterogeneity among studies, a fixed-effects model (the Mantel–Haenszel method) was used [27]. Otherwise, if the P-value < 0.05, the random-effects model (the DerSimonian and Laird method) was used [28]. The Z test was used to determine the significance of the combined OR and P < 0.05 was considered statistically significant.

Sensitivity analyses to evaluate the possible biases of the results in our meta-analyses were also performed. In addition, we assessed the potential publication bias with funnel plots of the effect sizes versus the standard errors and identified the significant asymmetry by the Begg’s test. An asymmetric plot suggests possible publication bias. If the P value < 0.05, the publication bias was considered significantly.

## Results

### Studies selected

**Figure 1:**
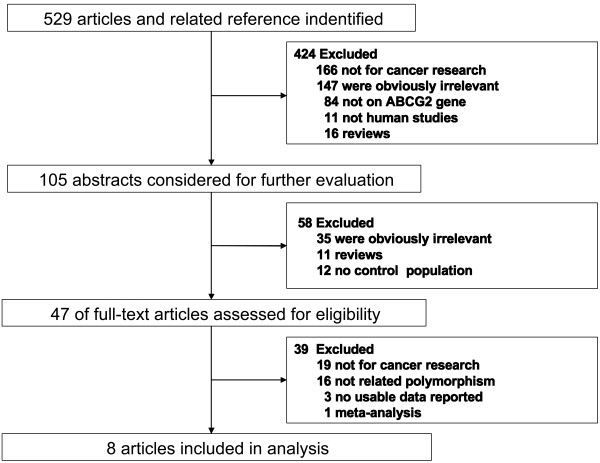
Flow diagram of included and excluded studies.

The meta-analysis included 8 eligible articles comprising 9 case–control studies and 1 case-cohort study, for a total of 3593 cases and 5875 controls. The study selection procedure is showed in Figure 1 and the study characteristics are displayed in Table 1[29-36]. Among all 10 studies, 2 focused only on colorectal cancer, 3 focused on lymphoma, 3 focused on leukemia and 2 focused on other cancers. Among the 10 eligible studies, 8 studies were conducted in Caucasian populations, and 2 studies were conducted in Asian populations. The control sources were population-based in 3 studies, hospital-based in 6 studies and both population-based and hospital-based in 1 study. The genotype frequency data for the C421A polymorphism in ABCG2 were extracted from all the eligible studies, and the distributions of the genotypes in the control populations were consistent with Hardy-Weinberg equilibrium in all of the studies. 

**Table 1:** Characteristics of studies included in the meta-analysis

**First author**	**Year**	**Country**	**Ethnicity**	**Cancer type**	**Source of controls**	**Cases**	**Controls**	**Case**					**Control**	**HWE**
								CC	CA	AA	CC	CA	AA	
Andersen[29]	2009	Denmark	Caucasian	Colorectal cancer	PB	359	765	296	58	5	592	161	12	0.782
Campa[31]	2008	Germany	Caucasian	Colorectal cancer	HB	582	517	472	103	7	409	104	4	0.347
Campa[30]	2011	Germany	Caucasian	Lymphoma	HB	1067	1196	898	158	11	957	229	10	0.359
Campa[30]	2011	Germany	Caucasian	Lymphoma	HB	259	1156	224	33	2	921	221	14	0.856
Hu[33]	2007	China	Asian	Lymphoma	MIXED	156	376	60	80	16	181	162	33	0.703
Campa[30]	2011	Germany	Caucasian	Leukemia	HB	321	1196	284	33	4	957	229	10	0.359
Muller[35]	2008	Germany	Caucasian	Leukemia	HB	110	179	100	10	0	160	18	1	0.532
Semsei[36]	2008	Hungary	Caucasian	Leukemia	HB	369	149	294	72	3	121	28	0	0.206
Korenaga[34]	2005	Japan	Asian	Renal cell carcinoma	PB	200	200	124	60	16	92	91	17	0.405
Gardner[32]	2008	America	Caucasian	Prostate cancer	PB	170	141	142	27	1	111	27	3	0.384

PB: population based; HB: hospital based; MIXED: population based and hospital based.

### Quantitative data synthesis

**Figure 2:**
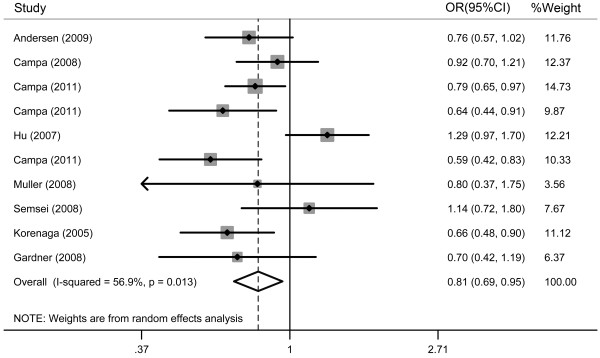
**Forest plot of overall cancer risk associated with the ABCG2 C421A polymorphism: A allele versus C allele.** (additive, random-effects model).

Overall, there was evidence for an association between decreased cancer risk and the variant genotypes in different genetic models and the results were listed in Table 2 and Figure 2. A significant decreased association between the ABCG2 C421A genotype and cancer risk was observed in our meta-analysis of the 10 studies (heterozygote model-AC versus CC: OR = 0.759, 95%CI = 0.620-0.930; dominant effects model-AA/AC versus CC: OR = 0.771, 95%CI = 0.634-0.938; additive effects model-A allele versus C allele: OR = 0.809, 95%CI = 0.687-0.952).

**Table 2:** Total and stratified analysis of ABCG2 C421A polymorphism on cancer risk

**Variables**	AA versus CC		AC versus CC		A versus C		Recessive model		Dominant model	
	OR(95%CI)	*P*^a^	OR(95%CI)	*P*^a^	OR(95%CI)	*P*^a^	OR(95%CI)	*P*^a^	OR(95%CI)	*P*^a^
Total	1.021(0.730-1.428)	0.784	**0.759(0.620-0.930)**^***b***^	0.004	**0.809(0.687-0.952)**^***b***^	0.013	1.067(0.768-1.482)	0.935	**0.771(0.634**-**0.938)**^*b*^	0.005
**Cancer type**										
colorectal cancer	1.073(0.491-2.344)	0.470	**0.791(0.633-0.988)**	0.445	0.840(0.688-1.025)	0.353	1.127(0.516-2.462)	0.494	0.806 (0.649-1.001)	0.388
lymphoma	1.200(0.736-1.958)	0.541	0.867(0.544-1.381) ^***b***^	0.003	0.874(0.600-1.273) ^***b***^	0.004	1.113 (0.692-1.791)	0.724	0.875(0.552-1.385) ^*b*^	0.002
leukemia	1.371(0.507-3.704)	0.750	0.744(0.426-1.299) ^***b***^	0.038	**0.741(0.577-0.951)**	0.072	1.474(0.543-4.002)	0.752	0.762(0.451-1.290) ^*b*^	0.049
other	0.627(0.314-1.251)	0.419	**0.574(0.407-0.808)**	0.205	**0.669(0.511-0.876)**	0.827	0.822(0.420-1.607)	0.309	**0.582 (0.420-0.807)**	0.347
**Source of control**										
Population based	0.684(0.383-1.223)	0.661	**0.647(0.510-0.821)**	0.289	**0.710(0.582-0.867)**	0.800	0.840(0.477-1.480)	0.594	**0.654(0.520-0.823)**	0.406
Hospital based	1.155(0.683-1.954)	0.897	**0.721(0.627**-**0.829)**	0.127	**0.780(0.687-0.885)**	0.158	1.225(0.724-2.075)	0.904	**0.739(0.645-0.847)**	0.133
HB + PB	-				-		-		-	
**Ethnicity**										
Asian	1.040(0.633-1.707)	0.143	0.856(0.287-2.549) ^***b***^	0.000	0.923(0.478-1.784)^*b*^	0.002	1.069(0.666-1.715)	0.623	0.882(0.317-2.457) ^*b*^	0.000
Caucasian	1.006(0.637-1.587)	0.846	**0.723(0.638-0.820)**	0.280	**0.773( 0.690-0.866)**	0.320	1.065(0.675-1.682)	0.849	**0.737(0.652-0.833)**	0.293

^a^P value of Q-test for heterogeneity test.

^b^Random-effects model was used when P value for heterogeneity test <0.05; otherwise, fix-effects model was used.

The same association was discovered in the subgroup analyses. In the subgroup analysis by cancer types, the ABCG2 C421A genotype significantly reduced the risk of leukemia and other cancers in additive model (OR = 0.741, 95%CI = 0.577-0.951; OR = 0.669, 95%CI = 0.511-0.876). Meanwhile, the variant heterozygote homozygote genotype AC was associated with significantly decreased colorectal cancer risk (heterozygote model-AC versus CC:OR = 0.791, 95%CI = 0.633-0.988) compared with the wild-type homozygote genotype CC; When the studies were stratified by the source of controls, associations were observed in both studies with population-based controls (OR = 0.710, 95%CI = 0.582-0.867) and those with hospital-based controls (OR = 0.780, 95%CI = 0.687-0.885). In the dominant effects model and heterozygote model, this association was also present and the result was listed in Table 2; In the analysis stratified by population, the C421A polymorphism in ABCG2 was significantly correlated with cancer risk in Caucasian populations (OR = 0.773, 95%CI = 0.690-0.866) but not in Asian populations (OR = 0.923, 95%CI = 0.478-1.784) populations in additive genetic model, however, in the dominant effect model and heterozygote model, no significant association between the ABCG2 polymorphism and low cancer risk was found in Asian in comparison to in Caucasian and the result was presented listed in Table 2.

### Heterogeneity analysis

There was significant heterogeneity among studies in the additive model, dominant effect model and heterozygote model (2AA + AC versus 2CC + AC, AA + AC versus CC, AC versus CC) of the C421A polymorphism in ABCG2. However, in the other model comparisons (AA versus CC, AA versus AC/CC), heterogeneity was not found (Table 2). We assessed additive model comparison, heterozygote comparison and dominant model comparison by tumor type, ethnicity, publication year, control source, sample size and HWE in controls. However, we did not observe any contribution to the substantial heterogeneity.

### Sensitivity analyses

**Figure 3:**
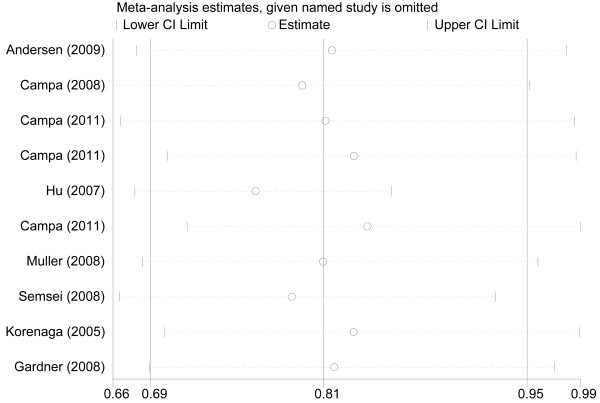
**Influence analysis for A allele versus C allele in the overall meta-analysis.** This figure illustrates the influence of individual studies on the summary OR. The middle vertical axis shows the overall OR and the two vertical axes indicate the 95% CI. Results were computed by omitting each study (left column) in turn. Meta-analysis random-effects estimates were used.

We performed sensitivity analyses by sequentially removing individual eligible study (Figure 3). The results indicated that the overall significance of the ORs was not altered by any single study in the genetic models for the C421A polymorphism in ABCG2 and cancer susceptibility which suggested the stability and liability of our overall results.

### Publication bias

**Figure 4:**
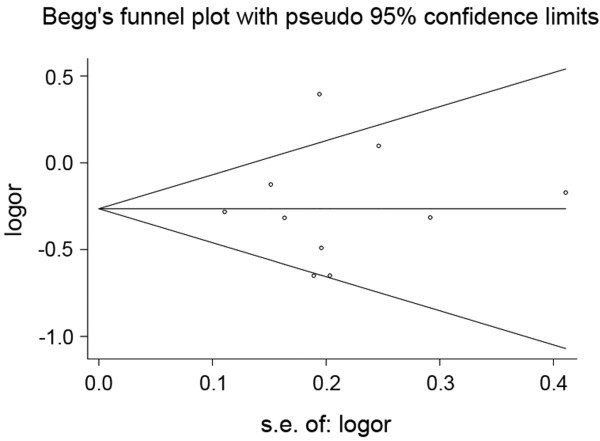
**Begg’s funnel plot for publication bias test (AA/AC VS CC); each point represents a separate study for the indicated association.** Log(OR): natural logarithm of OR. Horizontal line represents size of effect.

We assessed the potential publication biases of the included studies using the Begg’s funnel plot and Egger’s test (Figure 4), the result showed no significant evidence of publication bias (t = 0.15, P = 0.884 for dominant effects model**).**

## Discussion

Carcinogenesis is a complex process that involves both genetic and environmental factors and their interactions [1]. Since the genetic factors such as single nucleotide substitutions and gross chromosomal aberrations were considered to make more contribution to tumorigenesis, numerous studies focused on the candidate-polymorphism approach notably increased the number of associations between polymorphism and cancer risk [37]. Recently, increasing attention has been paid to the relationships between genetic variants such as SNPs and cancer risk. Plentiful epidemiological evidence demonstrated that the process of detoxification and elimination of xenobiotics was involved in the development of cancers [14,16,17].

The ABCG2 gene (also known as BCRP), located on chromosomal locus 4q22, encodes an ABC half-transporter protein that localizes to the plasma membrane. ABCG2 works as a multidrug efflux pump, influencing the metabolism of multiple substances including anticancer drugs and carcinogenic xenobiotics [10-13]. The abnormal distribution of carcinogenic xenobiotics may increase the local carcinogen burden of specific cells and organelles [38] and cause tumorigenesis. To date, there have been a great deal of epidemiological studies to investigate the association between the C421 polymorphism in ABCG2 and the risk of various types of cancers, however, the exact relationship between cancer susceptibility and the ABCG2 C421A polymorphism remains unclear. To address this issue, we performed a synthetic analysis based on data collected from all studies that have investigated the relationship between this polymorphism and cancer risk.

Generally speaking, our meta-analysis, which included 3593 cases and 5875 controls, revealed a significant association between the ABCG2 C421A allele and decreased cancer risk in different genetic model (heterozygote model-AC versus CC: OR = 0.759, 95%CI = 0.620-0.930; dominant effects model-AA/AC versus CC: OR = 0.771, 95%CI = 0.634-0.938; additive effects model-A allele versus C allele: OR = 0.809, 95%CI = 0.687-0.952). This result provides convincing evidence that the C421A polymorphism in ABCG2 might protect against cancer development. Moreover, this effect persisted when the studies were stratified by ethnicity classification. When stratified by cancer, source of controls and ethnicity classification, the C421A polymorphism of ABCG2 was also an important protective factor against cancer development in the subgroups studied.

However, the studies published by Hu and Korenaga [33,34] were contradictory to ours, indicating that carriers of the A allele of ABCG2 C421A had an increased risk of cancer. Three explanations may have contributed to this disparity in results. One possible explanation could be that the different environmental factors or different sample size of the two studies may influence the function of the ABCG2 C421A for developing cancer. Another possible explanation for this observation was that gene-gene interactions and gene-environment interactions may be responsible for this discrepancy. Moreover, the overlapping function of the other ABC transporters may be involved in. However, the precise mechanism of the contradictory effect remains unsure, further studies may help to clarify this issue.

It is well known that four capital factors of genome-wide association studies (GWAS) consisting of models of the allelic architecture of common diseases, sample size, map density and sample-collection biases need to be taken into account in order to optimize the cost efficiency of identifying precise disease-susceptibility loci [39]. To validate the strong associations between the C421A polymorphism in ABCG2 and the cancer risk, Daniele Campa and his colleagues extracted date from two previous GWAS on CLL (chronic lymphocytic leukemia) [40,41]. Based on the genotyping information from them, allelic odds ratios were calculated to confirm the relationship between the genetic polymorphisms and CLL risk. Their results identified a statistically significant association between the risk of CLL and the ABCG2 C421A genotype [30]. Furthermore, these authors also performed a meta-analysis of 3 studies [30,40,41], which provided evidence of a significant association between decreased cancer risk and the ABCG2 C421A polymorphism. Our findings were consistent with these results [30].

Heterogeneity is a potential problem which might influence the interpretation of the results. In our meta-analysis, significant heterogeneity between studies was present in additive model, heterozygote model and dominant model (Table 2). The heterogeneity reduced or disappeared when the studies were stratified by cancer type, source of controls and ethnicity, however, we did not find adequate evidence to determine which of them contributed most to the substantial heterogeneity. The publication bias for the association between this polymorphism and cancer risk was not observed in our meta-analysis.

Some possible limitations of our meta-analysis should be acknowledged and taken into consideration. First, detailed information, such as the mean age and sex of the case and control populations, was not available in all of the selected studies, which limited further analyses. Second, the results may be influenced by the lack of observations regarding gene-gene and gene-environment interactions even different polymorphic loci of the same gene. Third, the conclusions had the possibility to be disturbed due to the existence of overlapping function of the other ABC transporters. Fourth, the numbers of published studies were not sufficiently enough for a comprehensive analysis on different types of cancer. For example, there were no published data for gastric cancer, nervous system neoplasm and lung cancer with association of ABCG2 C421A published up to now; we did not posses enough statistical power to detect the precise association. More studies are needed to explore the relationship between C421A polymorphism in ABCG2 and cancer risk. In spite of these potential limitations, our meta-analysis also has many advantages. Firstly, sufficient date was extracted form well-selected studies, providing good statistical power for this meta-analysis. Secondly, studies included in our meta-analysis contained available genotype frequency and the distribution of the genotypes in the control population of all the studies were consistent with Hardy-Weinberg equilibrium. Thirdly, no publication bias was detected among the pooled results.

## Conclusions

In conclusion, this meta-analysis demonstrates that the ABCG2 C421A polymorphism is associated with a decreased risk of cancer and is likely a protective factor against cancer development. However, further studies on the relationship between this polymorphism and cancer risk are warranted.

## Misc

Pin Chen and Lin Zhao are contributed equally

## Competing interests

The authors declare that there are no competing interests.

## Authors' contributions

PC participated in collection of data and manuscript preparation. LZ, PZ and HX performed the statistical analysis. PZ participated in study design and critically revised the manuscript. PZ and AL participated in study design and manuscript preparation All authors read and approved the final manuscript.

## Pre-publication history

The pre-publication history for this paper can be accessed here:

http://www.biomedcentral.com/1471-2407/12/383/prepub
